# Molecular genotyping of G6PD mutations and Duffy blood group in Afro-descendant communities from Brazilian Amazon

**DOI:** 10.1590/1678-4685-GMB-2017-0253

**Published:** 2018-11-29

**Authors:** Haiala S. Silva de Oliveira, Aylla N. Lima Martins da Silva, Gabriela Barreto Andrade, Karoline Coelho Gaia, Greice de Lemos Cardoso Costa, Ândrea K. Campos Ribeiro dos Santos, João Farias Guerreiro

**Affiliations:** ^1^ Laboratório de Genética Humana e Médica, Instituto de Ciências Biológicas, Universidade Federal do Pará, Belém, PA, Brazil

**Keywords:** G6PD mutations, Duffy blood group, Afro-descendants, Brazilian Amazon

## Abstract

Glucose-6-phosphate dehydrogenase deficiency (G6PDd) and Duffy-negative blood group are two red blood cells variants that confer protection against malaria. In this study, the distribution of the most common G6PD variants (G6PD*A-, GGPD*A and *G6PD Mediterranean*) and the major alleles of the Duffy blood group (FY*A, FY*B and FY*B ^*ES*^) were investigated in an Afro-descendant population from state of Pará, Brazilian Amazon. G6PD variants and Duffy blood group alleles were determined by TaqMan SNP genotyping assay. Overall, molecular genotyping revealed the presence of G6PD variants in 126 (24%) of the individuals studied (5% male and 19% female), and frequencies of the G6PD*A- and G6PD*A alleles were 0.061 and 0.104, respectively. Duffy blood group genotyping showed that 24.3% of people were Duffy-negative and 41.3% were heterozygous for FY*B ^*ES*^. The frequency of allele FY*B ^*ES*^ was 41.0%. The results emphasize the need to monitor G6PD deficiency for the use of primaquine in the routine care of the Afro-descendant communities of the Trombetas, Erepecuru and Cumná rivers, evaluating the risks of hemolytic crisis in case of recurrence of malaria in the region. In addition, the possible greater protection against malaria conferred by these erythrocyte polymorphisms deserves to be better investigated and explored among these Afro-descendants.

## Introduction

Deficiency of glucose-6-phosphate dehydrogenase (G6PDd) and Duffy-negative blood group are two red blood cells variants that exhibit pattern of population distribution strongly influenced by natural selection by malaria. These variants are two well-known hereditary alterations, in addition to hemoglobin disorders and immunogenic variants, conferring protection against malaria, commonly found worldwide in areas inhabited by populations historically exposed to endemic malaria, including Africa, Mediterranean Europe, Southeast Asia and Latin America (review in Monteiro *et al.*, 2014).

G6PDd is primarily associated with protection against falciparum malaria (Ruwende *et al.*, 1995), but a protective effect of G6PDd has also been shown against malaria episodes caused by *Plasmodium vivax* (Leslie *et al.*, 2010, Santana *et al.*, 2013). Besides the protective effects against malaria infection, hemolysis induced by antimalarial drugs (primaquine) in G6PDd individuals is of major public health importance. The gene encoding G6PD, situated near the telomeric region of the long arm of the X chromosome (band Xq28), consists of 13 axons with a length of 18 kb (Pai *et al.*, 1980). Over 400 biochemical variants have been described and at least 217 mutations were identified in the G6PD gene. Not all mutations are polymorphic and of public health importance, and many appear only sporadically within populations. Almost half of the mutations identified are associated with the most serious clinical phenotypes and are very rare (Howes *et al.*, 2013). The variants are subdivided into five classes based on the functional severity of the deficiency: Class I, deficient variants associated with non-spherocytic hemolytic anemia; Class II, variants with severe deficiency (less than 10% of activity); Class III, variants with moderate deficiency (10% to 60% of activity); Class IV, variants with normal or slightly decreased activity (60% to 100%); Class V, variants with increased enzymatic activity. Clinically important variants are those of classes II and III because they are more common than those of class I, which are sporadic (Yoshida *et al.*, 1971; WHO Working Group, 1989).

Among all the known deficiencies, the G6PD*A and G6PD*Med (G6PD Mediterranean) variants are the most frequent in certain populations and are the main responsible for the occurrence of haemolytic events. The G6PD*A- variant predominates in sub-Saharan Africa where it affects 15 to 20% of the African population (review in Howes *et al.*, 2013). Until recently the G6PD*A- variant, a Class III type, was considered homogeneous when studied biochemically, but with the advent of molecular biology methods, DNA-level investigation has shown that this variant is heterogeneous. All types of G6PD*A- have in common the rs1050829 (G6PD c.376T>C mutation), that characterizes G6PD*A variant (Takizawa *et al.*, 1987), in addition to a second mutation, more commonly the rs1050828 (G6PD c.202C>T) (Hirono and Beutler, 1988), or rs137852328 (c.680G>T) (Beutler *et al.*, 1989), or rs76723693 (c.968T>C) (Beutler *et al.*, 1989). The African variant G6PD*A (c.376T>C) has electrophoretic mobility similar to that of the A- variant, and normal or very-mild deficiency (Class IV) enzymatic activity (Takizawa *et al.*, 1987). This variant occurs in 20-30% of Africans (Beutler, 1996). The Mediterranean variant, rs5030868 (c.563C>T transition), a Class II variant (Vulliamy *et al.*, 1988), is found in southern Europe, the Middle East and in the Indian subcontinent (Beutler, 1996; Howes *et al.*, 2013).

In Latin America, low prevalence rates of G6PDd are documented in Argentina, Bolivia, Mexico, Peru and Uruguay, but studies from Curaçao, Ecuador, Jamaica, Saint Lucia, Suriname and Trinidad, as well as some areas of Brazil, Colombia and Cuba, have shown a high prevalence (>10%) of G6PDd (review in Howes *et al.*, 2013; Monteiro *et al.*, 2014; Gómez-Manzo *et al.*, 2016). G6PD*A– (376G/202A) is the variant most broadly distributed across Latin America, found in 81% of the deficient individuals surveyed (review in Monteiro *et al.*, 2014; Gómez-Manzo *et al.*, 2016).

The Duffy glycoprotein, also known as the Duffy antigen receptor for chemokines (DARC), is a transmembrane glycoprotein that functions as a chemokine transporter. It is also a receptor for *Plasmodium vivax* and *Plamodium knowlesi* and expresses the Duffy blood group antigens (Fy). The Duffy blood group locus, at position q21–q25 on chromosome 1 (Donahue *et al.*, 1968), is characterized by three main alleles: FY*A, FY*B and FY*B ^*ES*^. The FY*A and FY*B alleles are distinguished by a missense mutation, which results in a single amino acid difference and gives the common Fy(a-b-), Fy(a-b+) and Fy(a+b+) phenotypes (Chaudhuri *et al.*, 1995; Iwamoto *et al.*, 1995; Mallinson *et al.*, 1995; Tournamille *et al.*, 1995a). The FY*B ^*ES*^ allele, which corresponds to the Fy(a-b-) serological phenotype (i.e., the absence of Fy antigen), is due to a T-33C point mutation on the FY*B gene promoter, which abolishes the erythroid gene expression by disrupting a binding site for the GATA-1 erythroid transcription factor. This mutation results in the elimination of the transcription of FY mRNA in red blood cells (RBCs), but not in other cell types (Tournamille *et al.*, 1995b; Pogo and Chaudhuri, 2000). The same mutation associated with the FY*A variant (FY*A ^*ES*^ allele) was already identified at low frequencies in individuals living in a *P. vivax*-endemic region of Papua New Guinea (Zimmerman *et al.*, 1999; Kasehagen *et al.*, 2007).

The distribution of Duffy alleles reveal strong geographic patterns, particularly the distribution of the silent FY*B ^*ES*^ allele across sub-Saharan Africa. The FY*B ^*ES*^ allele is at or near fixation in most sub-Saharan African populations. This allele is also found at high frequencies across Madagascar and through the Arabian Peninsula (above 80% and 50%, respectively), and at median frequencies (5–20%) across India and up to 11% in South-East Asia. However, it is very rare outside Africa, the lowest frequencies of the FY*B ^*ES*^ allele being found in the Americas (review in Howes *et al.*, 2011). The pattern of allele frequencies at FY locus has been attributed to a positive natural selection, based on the observation that individuals homozygous for the FY*B ^*ES*^ allele are highly resistant to vivax malaria, since *P. vivax* requires the presence of Duffy antigen receptor for chemokines on the RBC surface to be able to invade cells and cause disease (Miller *et al.*, 1976, Livingstone, 1984; Tournamille *et al.*, 1995b; Hadley and Peiper, 1997).

In this study, the distribution of the most common variants of glucose-6-phosphate dehydrogenase, G6PD*A- (G202A and A376G alleles), G6PD*A (A376G allele) and G6PD*Med (C563T allele), and the major alleles of the blood system Duffy (FY*A, FY*B and FY*B ^*ES*^), RBC variants that confer protection against malaria, were investigated in Afro-descendant communities along the Trombetas, Erepecuru and Cuminá rivers, in the rural area of the municipality of Oriximiná, in the northeast of the state of Pará, in the Brazilian Amazon. Until recently, this region was considered endemic for malaria but, as in the state of Pará and Brazil in general, there was a substantial reduction in the incidence of microscopically confirmed malaria cases between 2000 and 2016, due to the intensification of malaria control and prevention through the National Malaria Control Program (PNCM), Brazilian Ministry of Health. (Sivep-Malária, 2017). Based on this scenario, the present study has bioanthropological purposes, addressing the genetic variability associated to genetic variants considered as markers of African ancestry. At the same time, knowledge of the frequencies of these variants will allow assessing the risks of hemolytic crisis triggered by the use of primaquine in the treatment of malaria, as well as contribute to clarify aspects of the epidemiology of transmission of this pathology, with a description of biological factors of the human host that participate in malaria protection mechanisms.

## Subjects and Methods

### Sample collection

**Figure 1:**
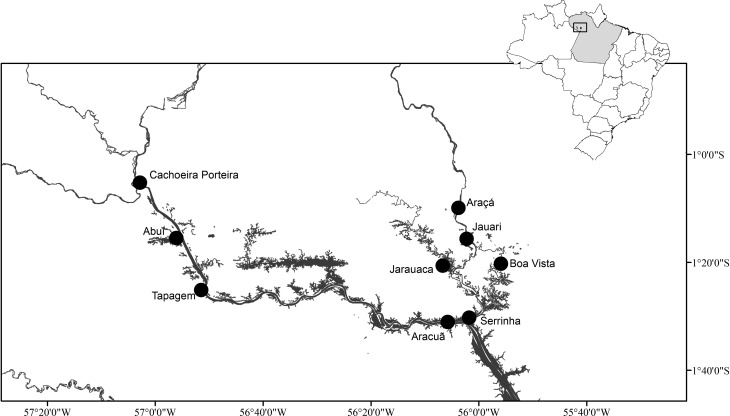
Geographical locations of Afro-descendant communities in the municipality of Oriximiná, northeast of the state of Pará.

Study protocol and sample collection were approved by the Ethics Committee on Human Research at the Institute of Health Sciences of the Federal University of Pará. This study was carried out in Afro-descendant communities along the Trombetas, Erepecuru and Cuminá rivers, a rural area in the municipality of Oriximiná, in the northeast of the state of Pará, in the Brazilian Amazon (Figure 1). Venous blood samples were collected in tubes containing EDTA, after informed consent, in a cross-sectional study conducted in these communities in 2011. Blood samples were collected from 594 individuals, aged between 15 and 70 years (mean age 35 years), 54.7% females. Two hundred and seventeen infividuals were from communities along the Trombetas River (Arancuan, Tapagem, Abuí and Cachoeira Porteira), a population estimated at 870 people, and 377 were from communities along the Erepecuru and Cuminá Rivers (Serrinha, Jauari, Araçá de Fora, Jarauacá and Boa Vista do Cuminá), with a total population of about 1320 people. These communities, known in the region as the “Blacks of the Trombetas”, are remnants of a *quilombo* created in 1820 under the leadership of the slave Atanásio, and by 1823 it had a population of about 2,000 runaway slaves when it was destroyed by the Portuguese (Klein, 1986).

### DNA extraction

DNA was extracted from 300 mL of EDTA-treated blood using the NeoIsoColumn kit (One Lambda Inc., San Diego, CA, USA) according to the manufacturer’s instructions. DNA was eluted in 200 μL of elution buffer (provided with the kit).

### G6PD genotyping

The single nucleotide polymorphisms (SNPs) rs1050829 (G6PD c.376T>C), rs1050828 (G6PD c.202C>T) and rs5030868 (c.563C>T) were investigated by a TaqMan SNP genotyping assay (Applied BioSystems, Foster City, CA, USA) according to the manufacturer’s instructions. Pre-designed probes were ordered for the genotyping analyses. About 10–50 ng of DNA were amplified with 5 μL of 2X TaqMan Universal PCR master mix, 0.5 μL of 40X primer and TaqMan probe dye mix. Cycling conditions were 10 min at 95 °C, followed by 40 cycles of 15 s at 92 °C and 1 min at 60 °C. Allelic discrimination was performed on an Applied BioSystems Real Time-PCR system.

### Duffy blood group genotyping

The samples were genotyped using primers (forward and reverse) and TaqMan fluorescence-labeled probes for real-time PCR designed using File Builder 3.1, Applied Biosystems, to genotype FY*A (G125A, rs2814778), FY*B (G125A, rs12075) and FY*B ^*ES*^ (T-33C, rs863002) alleles at the Duffy blood group locus. Allelic discrimination was also performed on a real-time PCR platform (Applied Biosystems 7500 analytical PCR system (SDS version 1.7).

Double heterozygotes in the promoter region (T-33C) and in the coding sequence (G125A) were also genotyped using an allele-specific PCR technique described by Olsson *et al.* (1998). Amplification was performed for each subject with sense primers corresponding to the normal and GATA-1-mutated promoter sequence combined with antisense primers that discriminate the FY*A and FY*B alleles in four different combinations of primers. PCR products were separated electrophoretically in 1.5% agarose gels at 150 V for 30 min and visualized with SYBR® Safe DNA gel stain under UV excitation. *G6PD* allele frequencies were calculated by direct counting and expected Hardy-Weinberg values were estimated using Chi-squared method.

## Results

### G6PD genotypes and allele frequencies

The observed G6PD genotypes distribution and the estimated G6PD allele frequencies are shown in Table 1. The G6PD*A- (376G/202A) variant was identified in 10 (1.9%) hemizygous males and in 36 (6.5%) females (32 heterozygous and 4 homozygous). The G6PD*A (376G) variant was found in 17 (3.2%) hemizygous males and in 63 (11.6%) females (55 heterozygous and 8 homozygous). The Mediterranean variant (G6PD c.563C>T), was not detected. Overall, 5% of the males and 19% of the females presented a G6PD variant, and the global frequencies of the G6PD*B, G6PD*A- and G6PD*A alleles were 0.835, 0.061 and 0.104, respectively. No deviation from Hardy–Weinberg equilibrium was observed in the sample studied (*p*=0.22).

**Table 1:** G6PD allele frequencies in Afro-descendant communities from Brazilian Amazon.

Region	Gender	N (%)	Allele		
			G6PD*B (%)	G6PD*A- (%)	G6PD*A (%)
Erepecuru river	Male	138 (40.8)	0^.^877	0^.^014	0^.^109
	Female	200 (59.2)	0^.^835	0^.^040	0^.^125
	Total	338 (100.0)	0^.^846	0^.^033	0^.^121
Trombetas river	Male	61 (33.2)	0^.^836	0^.^131	0^.^033
	Female	123 (66.8)	0^.^817	0^.^098	0^.^085
	Total	184 (100.0)	0^.^821	0^.^104	0^.^075
Overall	Male	199 (38.1)	0^.^864	0^.^050	0^.^085
	Female	323 (61.9)	0^.^829	0^.^065	0^.^106
	Total	522 (100.0)	0^.^835	0^.^061	0^.^104

When the distribution of the G6PD variants in the communities was analyzed according to geographical location (Trombetas and Erepecuru rivers), it was possible to observe that in the Trombetas communities the G6PD*A- variant is more common than the G6PD*A variant (10%, 4% and 7.5%, respectively), contrary to what was observed in the Erepecuru communities where the G6PD*A variant predominates. Overall, the communities of Erepecuru exhibited a lower frequency of the A- variant (3.3% versus 10.2%) and a higher frequency of variant A (12.1% versus 7.5%) when compared to the Trombetas communities. No deviations from Hardy-Weinberg equilibrium were observed in the studied communities (Erepecuru, *p*=0.07; Trombetas, *p*=0.12).

### Duffy blood group genotypes and allele frequencies

Overall, 20.4% (121/594) individuals were genotyped as Duffy-negative (FY*BES/*B^ES^) and 41,3% (254/594) were heterozygous for the FY*B ^ES^ allele (26.0% *FYA/FYB* ^*ES*^ and 15.3% *FYB/FYB* ^*ES*^). In the total sample, the frequency of allele FY*B ^*ES*^ was the highest (41.0%) (Table 2). The genotype frequencies observed in the communities grouped by geographical location revealed a similar profile, with predominance of the FY*B ^*ES*^ /FY*B ^*ES*^ genotype in both regions (17.9% in the Erepecuru river and 25.1% in the Trombetas river) and high heterozygote frequencies for the FY*B ^*ES*^ allele (40.4% in the Erepecuru River and 41.3% in the Trombetas River). The FY*B ^*ES*^ allele was also the most common one in the communities of both regions, but the frequency observed in the Trombetas River (47.5%) was higher than that found in the Erepecuru River (37.6%). Allele frequencies were in Hardy-Weinberg equilibrium both in the global sample and in the communities grouped by geographical location (Trombetas and Erepecuru), with *p*=0.22.

**Table 2:** Duffy allele frequencies in Afro-descendant communities from Brazilian Amazon.

Region	N	Allele		
		FY*A	FY*B	FY*B ^*ES*^
Erepecuru river	391	0.352	0.272	0.376
Trombetas river	203	0.291	0.234	0.475
Overall	594	0.331	0.259	0.410

## Discusssion

### G6PD variants

The results obtained in this study with Afro-descendants of Pará in the Brazilian Amazon, the “Blacks of the Trombetas”, can be considered as expected for a population of predominantly African origin, with some degree of local differentiation and admixture with people of Caucasian and Amerindian ancestry. The G6PD A- (376 A>G and 202 G>A) and A (376 A>G mutation) variants, found here at frequencies of 0.061 and 0.104, respectively, are the most common variants in Africa, particularly in sub-Saharan Africa, where they reach frequencies between 15% and 30%. The frequencies observed for these variants among the Afrodescendants were lower than those described for most of the sub-Saharan African populations (Carter *et al.*, 2011; Howes *et al.*, 2013), but are similar to those found by Millimono *et al.* (2012) in the Republic of Guinea, West Africa, as follows: G6PD*A- (A376G/G202A), 5.7% and G6PD*A (A376G), 17.6%.

Comparisons of the distribution of the G6PD variants in Afro-descendants studied here with those observed in other Brazilian populations can be made with other studies carried out in Brazil using the same approach, namely the use of molecular biology methods for genotyping, regardless of previous enzymatic activity screening. The frequency of the G6PD*A- variant among the Afro-descendants studied here (6.1%) is somewhat higher than that observed by Santana *et al.* (2013) among males from the population of Manaus, state of Amazonas (3.8%) and by Dombrowski *et al.* (2017) in males from the Juruá valley, state of Acre, northern Brazil. It is also higher than the frequency found by Castro *et al.* (2007) in newborns from Porto Alegre, southern Brazil (2.9%), but is similar to those found in Campinas (6.1%) and São Paulo (5.8%), southeastern Brazil, by Mezzacappa *et al.* (2010) and Oliveira *et al.* (2009), respectively. On the other hand, the frequency of G6PD*A- in the current study is lower than the frequency of 8.2% reported by Moura-Neto *et al.* (2008) for the population of Salvador, Bahia, northeastern Brazil, a population of predominantly African origin (Santos *et al.*, 2016).

The other variant found among Afro-descendants, the African variant G6PD*A, which exhibited an unexpected frequency of 10.4%, was found in the population of Salvador, Bahia, with a frequency of 3.1% (Moura-Neto *et al.*, 2008) and in a sample of Black males from Rio Grande do Sul, with a frequency of 8% (Weimer *et al.*, 1981). This finding may be a particular feature of the Afro-descendant population studied here, particularly those from Erepecuru River, reflecting the origin of the African slaves who founded these communities. Alternatively, the absence of the G6PD*A variant in other populations may be attributed to the fact that most studies investigating G6PD mutations are carried out only in subjects with enzyme deficiency, and so the variant would be less likely to be detect since it exhibits normal activity or a very-mild deficiency. Therefore, the search for G6PD variants only in individuals with enzyme deficiency appears to hide a genetic variability revealed only in population-based studies. On the other hand, the absence of the Mediterranean variant among the Afro-descendants studied is an expected result, given the predominantly African origin of the communities, and the absence of evidence of miscegenation with individuals of Italian, Greek, Arab or Jewish origin, in whom this allele is more common. In Brazil, this variant was found in the cities of Campinas (Saad *et al.*, 1997) and Araraquara (Ferreira *et al..* 2014), both in the state of São Paulo, southeastern Brazil, with frequencies of 3.0%, and in Caucasian males from Porto Alegre, state of Rio Grande do Sul, with a frequency of 8.0% (Weimer *et al.*, 1981). The variant was also found in the population of Manaus with a frequency of 1.0% (Santana *et al.*, 2013) attributed to the contribution of Arabs and Jews in the population of the city of Manaus in the state of Amazonas.

Intracontinental comparisons (Latin America) show that the results observed in the “Blacks of the Trombetas” are more similar to those reported by Petit *et al.* (2016) in people from French Guiana, where the variants G6PD*A- and G6PD*A were found at frequencies of 0.11 and 0.10, respectively. The frequency of G6PD*A- found in the current study (0.06) is similar to the ones found in Colombia and Honduras (0.09), but is higher than that reported for Venezuelans (0.017). The African G6PD*A variant, however, has been found at lower frequencies in these three Latin American countries: 0.02 in Colombia, 0.03 in Honduras, and 0.002 in Venezuela (Valencia *et al.*, 2016, Zuñiga *et al.*, 2015, Vizzi *et al.*, 2016).

The results obtained in the present study emphasize the need to use appropriate G6PDd diagnostic methods in the routine care of vivax malaria in the Amazon region, especially in Afro-descendant communities, to avoid hemolysis induced by treatment with primaquine in individuals genetically deficient for G6PD. Primaquine (PQ) is an 8-aminoquinoline considered the most effective drug against the latent hepatic stages of *P. vivax*, preventing clinical relapses, but is also highly active against gametocytes of all species of human malaria, preventing further transmission of parasites for mosquitoes (WHO, 2015). Therefore, primaquine is used for both *P. vivax* and *P. falciparum* treatment, but the dosage for *P. vivax* malaria is much higher than the single low dose recommended by WHO (0.25 mg base/kg) to block transmission of *P. falciparum* malaria, being associated with a considerably lower risk of haemolytic toxicity. The test for the reduction of methemoglobin (Brewer *et al.*, 1962), due to its high sensitivity and low cost, may also be used for monitoring the presence of G6PDd, although a rapid diagnostic test (RDT) to detect G6PDd in the Brazilian Amazon is already available and is a good cost-effective strategy for diagnosing G6PDd. Based on this monitoring, it is recommended that in patients with a diagnosis of G6PDd, treatment with primaquine should be adjusted for intermittent administration of 0.75 mg/kg weekly for eight weeks under medical supervision in order to reduce the risk of hemolysis (Silva *et al.*, 2004; Brito *et al.*, 2016; Peixoto *et al.*, 2016).

### Duffy blood group alleles

The distributions of Duffy blood group genotypes and alleles among the “Blacks of the Trombetas” were also consistent with the predominantly African origin of the population. The observed frequencies of the Duffy-negative genotype (20%) and FY*B ^*ES*^ allele (41%) were slightly different from those previously described in Afro-descendant communities from the states of Pará and Amapá (Perna *et al.*, 2007), where frequencies of the Duffy-negative genotype ranged from 32.3% to 58.8%, and frequencies of the FY*B ^*ES*^ allele ranged from 56.4% to 72.2%. On the other hand, the frequencies found in Afro-descendants were higher than those reported for Amazonian riverine communities, where frequencies of the Duffy-negative genotype are usually low, varying from 3% to 8%, and the mean frequency of FY*B ^*ES*^ is around 5% (Cavasini *et al.*, 2007).

Considering that the Duffy-negative blood group is a well-documented protection factor against *P. vivax* malaria and that there is also evidence that G6PDd protects against *P. vivax* (Louicharoen *et al.*, 2009; Leslie *et al.*, 2010; Santana *et al.*, 2013), it is possible to consider that the co-occurrence of the G6PD variant and the FY*B ^*ES*^ allele in polymorphic frequencies in the “Trombetas Blacks may confer greater protection against malaria in this population. Indeed, 24% of the individuals presented a G6PD variant (1.9% hemizygous, 0.9% homozygous and 5.7% heterozygous), and 61.7% presented the FY*B ^*ES*^ allele (41.3% heterozygotes and 20, 4% homozygotes). In addition, an earlier study in these communities identified the HBB*S gene, which codes for hemoglobin S, being the first of the structural variants of hemoglobin associated with malaria protection, with a frequency of 6% (Schneider *et al.*, 1987).

The greater protection against malaria conferred by these erythrocyte polymorphisms deserves to be better investigated and explored. Until the 1990s the region of Trombetas, where *quilombolas* and riverine people live, was considered endemic for malaria, accounting for most cases of malaria recorded in the municipality of Oriximiná. From the year 2000, however, there was a significant decrease in the number of cases in the region. This was initially due to the implementation of a malaria control project in the Trombetas river region, by Mineração Rio do Norte S.A., in collaboration with the Endemism Sector of the Municipality of Oriximiná, and later with support from the Brazilian Ministry of Health through the National Program for Malaria Control (PNCM) that started in 2003. With these actions, the municipality of Oriximiná, which was considered a medium risk region for malaria, is now considered low risk, based on the annual parasite index (IPA) (Sousa *et al.*, 2015). However, data from the Brazilian Ministry of Health indicate that the number of malaria cases has increased again in Brazil, after years of decline, particularly in the state of Pará, where the number of cases in 2017 has more than doubled compared to 2016. Most likely, there will also be an increase in the number of malaria cases in the region where Afro-descendants live (Trombetas, Erepecuru and Cuminá rivers), and procedures for prompt diagnosis and treatment of the disease will become necessary again. Thus, monitoring of G6PDd in cases of primaquine use should play an important role in the routine care of Afro-descendant communities to assess the risks of hemolytic crisis in the case of malaria recurrence.
